# Comparisons of reproductive function and fatty acid fillet quality between triploid and diploid farm Atlantic salmon (*Salmo salar*)

**DOI:** 10.1098/rsos.180493

**Published:** 2018-08-15

**Authors:** D. S. Murray, M. J. Kainz, L. Hebberecht, K. R. Sales, K. Hindar, M. J. G. Gage

**Affiliations:** 1 School of Biological Sciences, University of East Anglia, Norwich Research Park, Norwich NR4 7TJ, UK; 2 WasserCluster – Biologische Station Lunz, 3929 Lunz am See, Austria; 3 Department of Zoology, University of Cambridge, Downing Street, Cambridge CB2 3EJ, UK; 4 Norwegian Institute for Nature Research (NINA), NO-7485 Trondheim, Norway

**Keywords:** aquaculture, nutrition, polyploidy, sustainability, sterility, sperm

## Abstract

Triploidy could prevent escaped farm salmon breeding in the wild, while also improving nutrient quality within farmed fillets. Despite these potential advantages, triploid Atlantic salmon have not been widely used in aquaculture, and their reproductive function has yet to be fully evaluated. Here, we compare reproductive function and fillet composition between triploid and diploid farm salmon under standard aquaculture rearing conditions. We show that female triploids are sterile and do not develop gonads. By contrast, males produce large numbers of motile spermatozoa capable of fertilizing wild salmon eggs. However, compared with diploids, reproductive development and survival rates of eggs fertilized by triploid males were significantly reduced, with less than 1% of eggs sired by triploid males reaching late-eyed stages of development. Analyses of fillets showed that total lipid and fatty acid quantities were significantly lower in triploid than in diploid Atlantic salmon fillets. However, when fatty acids were normalized to total lipid content, triploid fillets had significantly higher relative levels of important omega-3 long-chain polyunsaturated fatty acids. Our results show that: (i) escaped triploid farm salmon are very unlikely to reproduce in the wild and (ii) if able to match diploid fillet lipid content, triploid farm salmon could achieve better fillet quality in terms of essential fatty acids.

## 1. Introduction

Atlantic salmon farming has grown hugely since the 1970s, and it is now a dominant activity in aquaculture with approximately 2 million metric tonnes of salmon being produced per year globally [1]. Growth in salmon farming will continue, with aquaculture expected to play a major role in satisfying escalating global demands for animal protein [1]. Farmed salmon, in particular, has become a premium food product, containing high levels of omega-3 long-chain polyunsaturated fatty acids (n-3 LC-PUFAs), widely recognized as essential nutrients for human health [2]. However, despite aquaculture's success in widening consumer access to these essential nutrients, growth within the salmon-farming industry has come with environmental impacts, including risks to the genetic integrity of wild stocks through farm escape and introgression [3], and use of unsustainable fishmeal and inefficient feed conversion [1]. In this study, we test a potential solution to prevent farm salmon breeding and introgression into wild gene pools, and examine whether this solution can also improve the efficiency of feed conversion.

Every year, hundreds of thousands of farm Atlantic salmon are reported to have escaped from their net pens into the wild [4,5]. While most of these salmon do not tolerate natural selection and disappear, there is evidence that farmed escapees survive in the wild, disperse significant distances, enter freshwater systems and breed successfully with wild Atlantic salmon [6]. Investigation of 147 salmon populations around Norway discovered significant farm salmon introgression in half of the populations surveyed [7]. For 109 rivers, the average level of farmed introgression was 6.4%, with a range between 0.0% and 42.2%. Additionally, 51 of these rivers showed significant farmed genetic introgression when compared with historical reference samples [7]. Because farm salmon have been under intense domestic selection since the early 1970s [8], they have altered morphological, physiological and behavioural phenotypes compared with wild fish [9]. Detailed experiments in wild systems have shown that offspring containing domesticated genes show much reduced fitness and survival compared with wild salmon equivalents [6]. Accordingly, when escaped farm salmon breed with wild fish, the resulting ‘hybrid’ offspring contain maladapted genomes compared with locally adapted wild strains, potentially leading to ‘genetic swamping’ and the potential for ecological destabilization [3]. Inactivating the reproductive potential of farm salmon escapes would solve this introgression problem, and triploid induction is one possible route to achieving that [10].

Most salmon currently produced by the aquaculture industry are diploid, possessing the normal two sets of chromosomes within their somatic cells. However, polyploidy can often be tolerated in fishes, even playing a key role in evolutionary diversification across several teleost families, including salmonids [11]. The aquaculture industry has been creating and using polyploid fish since the 1970s [12], and triploidy has been successfully induced in more than 30 species [13]. Pressure or temperature shock on recently fertilized eggs can be used to induce triploidy, and these relatively cheap and straightforward methods cause the retention and incorporation of the egg's second polar body into the triploid zygote [10]. Artificially produced triploids carrying three sets of chromosomes usually differ from diploids in three fundamental ways: (i) they are generally more heterozygous, (ii) they have larger but fewer cells in most tissues, and (iii) their gonadal development is often reduced, making adults infertile [14]. This latter impact on reproductive potential can therefore be used to prevent breeding by escaped farm fish [11], and this has been successfully applied to prevent introgression of farm-strain rainbow trout (*Oncorhynchus mykiss*) and grass carp (*Ctenopharyngodon idella*), following stocking into rivers and lakes for recreational fishing, and to control invasive aquatic weeds [15,16].

In addition to reproductive containment of escaped fish, triploidy could also improve the feed sustainability and nutritional quality of farmed fish, which has been steadily declining in Atlantic salmon farming since 2006 [1]. Sexual maturation, especially in salmonids, demands significant nutrient diversion into gonadal growth and gamete production [17]. During maturation, dietary lipids and fatty acids (FAs) are directed towards gonadogenesis, at the expense of investment in somatic tissue [17]. Previous studies have found that saturated fatty acids (SAFAs) are preferentially mobilized away from the muscle tissue during sexual maturation in farmed fish [18–20]. The requirement of n-3 LC-PUFAs in embryonic development can cause the mobilization of eicosapentaenoic acid (EPA) and docosahexaenoic acid (DHA) away from muscle tissues towards gonadal development [21], reducing the n-3 PUFA content in the fillet for human consumers [22]. If triploid fish downregulate investment into reproductive development, n-3 LC-PUFA levels could be retained in muscle tissues, increasing the nutritional quality of farmed Atlantic salmon for human consumers.

Given concerns in salmon aquaculture about introgression and feed sustainability, the dual aims of this study were to: (i) investigate how triploid status influences gonadal development and reproductive function in farm Atlantic salmon and (ii) assess the impacts of triploidy on nutritional quality of the fillet. Triploid induction in fishes usually disrupts gametogenesis, with sterility a common outcome [10]. However, triploidy effects are highly variable among species and, while triploid egg production is generally rudimentary in most taxa, triploid males can sometimes develop normal-sized testes with running milt [11]. Spermatogenesis is often disrupted in triploid males, resulting in lower numbers of, sometimes abnormal, sperm [11,13]. However, it is not a certainty that triploidy always induces sterility. Triploid male fertility is reported in rainbow trout (*O. mykiss*), Siamese fighting fish (*Betta splendens*) and grass carp (*C. idella*) [23]. In more detailed work with plaice (*Pleuronectes platessa*), triploid testicular development appeared normal and triploid males produced large numbers of motile sperm, which successfully fertilized eggs that developed to gastrulation [24]. In triploid tench (*Tinca tinca*), testicular development and sperm density were reduced [25]. However, triploid males produced motile sperm that were able to fertilize eggs, which subsequently produced relatively high hatching rates [25]. It is therefore essential to assess the reproductive development and function of each individual species before triploidy can be assumed to induce sterility.

In Atlantic salmon, triploidy can be readily induced [10]; however, no detailed experimental measures of reproductive development, sperm function and fertilization potential have been published that compare diploid and triploid farm salmon under controlled conditions. While it is known that triploid male salmon can develop relatively large testes, experimental reports on triploid male fertility are limited to testing with a single individual, which clearly had some fertility (not reported) because very low (1.6%) survival of this male's offspring was reported after hatch [26]. It is additionally important to verify the reproductive status of triploid salmon, because male Atlantic salmons exhibit normal spawning behaviour in enclosed streams, releasing milt and inducing diploid females to spawn [27], reinforcing the importance of verifying their reproductive status [27]. Although triploid males should produce aneuploid sperm, these could fertilize and ‘occupy’ eggs from wild salmon. Moreover, because triploidy can facilitate hybridization in otherwise reproductively isolated salmonids (e.g. triploid *S. salar* can hybridize with chum salmon, *Oncorhynchus keta*) [13], it is important to assess whether viable embryos can be produced in significant numbers in this autotetraploid species.

Research has already shown that, under optimized conditions, triploid salmon can perform as well as diploids throughout the farm cycle [28]. Therefore, in parallel with our experimental assessment of the reproductive function of triploid farm salmon, we also measure the nutritional quality of triploid adult fillets, comparing FA content against diploid equivalents from the same farm strain reared under similar conditions and fed an identical diet.

## 2. Material and methods

### 2.1. Fish husbandry and rearing conditions

Experiments were carried out through the 2015–2016 and 2016–2017 spawning seasons at the Norwegian Institute of Nature Research (NINA) Aquatic Research Station in Ims, Norway. Fish were maintained and handled according to standard hatchery protocols approved by the Norwegian Animal Research Authority. Ninth generation farmed diploid and triploid Atlantic salmon juveniles of the same AquaGen^©^ strain (hatchery-reared from Norway's major breeding programme, Kyrksæterøra) were reared in holding facilities at Ims from May 2013 until November 2016. Diploid and triploid fry were held in separate freshwater tanks until reaching 2–3 g in weight. Triploid and diploid salmon juveniles were then transferred to separate tanks containing 3–5‰ salt water, where they were maintained under standardized conditions throughout until each spawning season. For this study, salmon were sampled in November 2015 and November 2016, when fish were approximately 2 and 3 years old.

Thirty 2-year-old Atlantic salmon were used to compare primary reproductive development. Forty 3-year-old salmon were used to compare gamete production and quality. Numbers of 2-year-old fish sampled: *n* = 6 diploid males; *n* = 7 triploid males; *n* = 9 diploid females; *n* = 8 triploid females. Numbers of 3-year-old fish sampled: *n* = 10 diploid males; *n* = 10 triploid males; *n* = 10 diploid females; *n* = 10 triploid females. The experiment was conducted in a flow-through system containing four identical circular tanks (4000 l each) with a continuous supply of UV-filtered Imsa river water (approx. 100 l min^–1^). Wastewater was drained using a sink-hole covered by a 5 mm mesh screen. Fish were subjected to a natural photoperiod (latitude, 58.0333°N). A total of 600 juvenile diploid Atlantic salmon and the same number of triploid salmon were randomly distributed as 300 fish of mixed sexes per tank. Dissolved oxygen, pH and water temperature were recorded daily. Throughout the experiment, Atlantic salmon were exposed to natural (ambient) water temperature (2.9–15.3°C; mean = 7.9°C), dissolved oxygen (7.3–11.4 mg l^−1^; mean = 9.2 mg l^−1^) and approximately neutral pH values (6.7–7.7; mean = 7.4). Fish in replicate tanks were fed by a clockwork belt feeder (Dryden Aqua Ltd) over a 12 h feeding period. The daily feed ration was the same for all tanks and exceeded the recommended feeding rate for salmonids for the prevailing water conditions [29].

### 2.2. Sampling procedure

After 2 and 3 years, diploid and triploid adults were selected at random from two replicate treatment tanks and had their fork length (mm) and weight (g) recorded for the calculation of condition factor. The relationship between mass and length (condition factor (*C*)) was evaluated by Fulton's formula: (*C* = 100 (*W*/*L*^3^); *W* = weight and *L* = length) owing to significant relationships between this condition factor and energy density for both sexes of the River Imsa Atlantic salmon [19]. Fish were killed by a blow to the head and samples of muscle and gonadal tissue dissected and stored in plastic vials (50 ml). Muscle samples were obtained in an identical manner for all individuals by cutting a fillet from the fish that included both dorsal and ventral muscle tissue, and excluding any skin or bone in the sample. All muscle tissue samples were stored at −80°C and freeze-dried for 48 h before analysis. Gonadal tissue from male and female salmon was weighed and the gonadosomatic index (GSI) calculated [30]. Gonad tissue subsamples were stored in 1.5 ml Eppendorf tubes containing buffered neutral formalin (BNF) for fixation.

### 2.3. Confirmation of triploidy using microsatellites

To unequivocally confirm ploidy status using DNA, we used a panel of established microsatellites [4]. DNA was extracted from adult fish using fin tissue collected during sampling. A PCRbio Rapid Extraction Kit (PCR Biosystems, UK) was used to efficiently extract the DNA. In brief, a small amount of tissue (approx. 5 mm) from each individual was placed along with 100 µl of rapid extract buffer in a 1.5 ml Eppendorf tube and incubated at 75°C for 5 min, vortexed twice for 10 s and incubated again at 95°C for 10 min. Finally, 900 µl of ultra-pure H_2_O was added before further analysis.

Polymerase chain reaction (PCR) was carried out in 10 µl volume reaction multiplexes within 96-well plates containing 1 µl of DNA (unspecified concentration), 1 µl of PCR Mastermix and 1 µl of multiplex primers (forward labelled primers SSsp3016 (GenBank no. AY372820), SSsp2210, SSspG7, SSsp2201, SSsp1605, SSsp2216 [31], Ssa197, Ssa171, Ssa202 [32], SsaD157, SsaD486, SsaD144 [33], Ssa289, Ssa14 [34], SsaF43 [35], SsaOsl85 [36], MHC I [37], MHC II [38] and reverse primers). The PCR ran with an initial 3 min denaturation step at 94°C preceding 39 denaturing (94°C for 15 s), annealing (61°C for 15 s) and extension (72°C for 15 s) cycles. PCR products were run on an ABI3730 automated sequencer at the John Innes Centre and sized using 500LIZ size standard.

Triploid Atlantic salmon were confirmed using microsatellite DNA genotyping and the identification of three clearly identifiable alleles per locus. The number of identifiable alleles per locus was determined using the genotyping software GeneMapper v. 4.0 (Applied Biosystems, USA). To confirm triploidy, an individual fish was reported as triploid if it displayed three clear alleles at two or more genotyped loci [4]. All fish designated as triploid Atlantic salmon, by AquaGen, at the start of the experiment were confirmed as triploids using this method (electronic supplementary material, table S1).

### 2.4. Histological examination of gonadal tissues

For histological examination of 2- and 3-year-old Atlantic salmon, 15 gonadal tissue samples from male and female diploid and triploid Atlantic salmon (*n* = 30) were dissected and placed into an Eppendorf tube containing BNF for histological examination. Samples were dehydrated, cleared and impregnated with paraffin wax. The paraffin-embedded sections were cut at 5 µm using a Leica RM2125 RT microtome (www.leicabiosystems.com) and sections were mounted onto glass slides for staining with haematoxylin and eosin. The microscopic anatomy of the chorion was examined using a Leica DC480 light microscope (Leica Biosystems) at 40–400× magnifications to identify any differences in the gross anatomy between diploid and triploid Atlantic salmon. A standardized terminology for describing reproductive development in fishes was used [39].

### 2.5. Sperm collection and analyses

Gametes were stripped in November 2016 from ten 3-year-old diploid and ten 3-year-old triploid adult fish using standard hatchery procedures to collect free-running milt from the vent of the males, ensuring no contamination with water, mucus or urine [40,41]. The volume of milt produced was determined by transferring stripped milt to 20 ml measuring cylinders. Stripped sperm samples were processed immediately after volumetric analysis. Subsamples were diluted in salmonid extender solution (80 mM NaCl, 40 mM KCl, 1 mM CaCl_2_ and 20 mM Tris, adjusted to pH 9 [42], at a 1 : 100 ratio). This procedure pre-dilutes the semi-viscous semen, so that sperm are evenly distributed within the solution for full activation and counting. Comparative analysis of diploid and triploid sperm concentrations was conducted with minor modifications from those previously described [40]. In brief, sperm volume was assessed using a measuring cylinder and sperm concentration was evaluated by light microscopy (200×) using a haemocytometer and calculating the density of sperm produced per millilitre of milt from both diploid and triploid Atlantic salmon.

For computer-assisted sperm analysis (CASA), we ran two independent recordings and CASA for each male's sperm sample. Pearson correlation analyses showed significant repeatability between the two recordings for measures of sperm motility (*r*^2^ = 0.97, *p* < 0.001), velocity (*r*^2^ = 0.92, *p* < 0.001) and linearity (*r*^2^ = 0.95, *p* < 0.001). Sperm were activated with distilled water so that the final semen concentration was approximately 20 × 10^6^ sperm ml^−1^ in a final volume of 1 ml. Immediately after dilution and rapid mixing, 10 µl of semen sample was placed in a counting chamber (Spermtrack, Proiser, Spain). All activations and recordings of sperm motility and fertilization trials were performed at the natural river water temperature of 2–4°C, in a similar air temperature.

Sperm motility was assessed under a microscope (UOP, Tokio, Japan) equipped with a 10× negative-phase contrast objective and a Grasshopper2 digital camera (FLIR systems^®^, British Columbia, Canada). Images were captured and analysed using Image J (United States National Institutes of Health) and the Integrated System for Semen Analysis Software plugin [43]. Straight line velocity (VSL), curvilinear velocity (VCL), average path velocity (VAP), linearity (LIN = VSL/VCL), straightness (STR = VAP/VCL), beat-cross frequency (BCF), wobble (WOB), amplitude of lateral head displacement (ALH) and percentage of motile spermatozoa were all measured. However, the parameters suggested most useful for studying sperm motility in fish are percentage of motile sperm, VCL (velocity of sperm along the path trajectory) and LIN (a measure of sperm trajectory) [44,45]. CASA settings were: 25 frames s^−1^ for acquisition, VCL > 10 µm s^−1^ to classify a spermatozoon as motile and 5–80 µm^2^ for head area.

### 2.6. Egg development

Approximately 100–200 eggs each were collected from 10 female Atlantic salmon (wild strain salmon from the Figgjo river system, Norway) during November 2016 and were subdivided into two replicates. We used a split clutch design to control for between-female variability, and eggs were fertilized <1 min after stripping by a single male diploid or triploid salmon in either split clutch, respectively. Eggs were then water-hardened and placed into individual incubation trays. Incubation trays were constructed from plastic mesh (5 mm mesh diameter) wrapped round a solid square Perspex base and rim (10 × 10 × 15 cm). These trays were placed in a 400 l flow-through tank (10 l min^−1^). Eggs were checked every other day for mortality, and white/opaque dead eggs were recorded and removed from the incubation system. The experiment was terminated when all eggs reached the eyed stage of development in February, following fertilisations in the previous November, an average of three months following fertilization. Water temperature during egg incubation ranged between 3.1 and 9.9°C (mean = 8°C).

### 2.7. Dietary proximate analysis

A commercially available isocaloric fish feed was used to provide sufficient lipid and protein to meet somatic requirements for salmonids [46]. The gross nutrient composition of the diet was determined as described in table 1. Moisture was determined by drying to constant weight in an oven at 110°C for 24 h [47]. Sample weight was recorded before drying and after removal from the oven. This process was repeated at 1 h intervals until weight change was ≤5 mg. Total protein content in experimental diets was determined by a modified Bradford assay [48] and total lipids by solvent extraction and gravimetric determination [49]. Ash content was determined by placing pre-weighed diets in a muffle furnace at 550°C for 8 h or until white ash was obtained [47] and was subsequently weighed.

**Table 1.** Proximate composition of diet fed to both diploid and triploid Atlantic salmon (g/100 g of diet) (mean ± s.e.).

content	diet
protein	49.9 ± 2.1
lipid	32.5 ± 1.9
ash	9.2 ± 0.5
moisture	7.5 ± 0.8

### 2.8. Lipid extraction and fatty acid analysis

Muscle tissue was harvested and analysed from 15 diploid and 15 triploid Atlantic salmon. Total lipids from homogenized and freeze-dried muscle (25–35 mg) and dietary samples (electronic supplementary material, table S2) were analysed as by Heissenberger *et al*. [49]. In brief, samples were sonicated and vortexed in chloroform/methanol (2 : 1 by volume). Organic layers were removed and transferred into solvent-rinsed vials. For the gravimetric determination of total lipid contents (i.e. mg lipids/g dry weight (dw)), duplicate subsamples (100 µl) of the extracts were evaporated and weighed. Fatty acids were derivatized to obtain fatty acid methyl esters (FAMEs) using toluene and sulfuric acid–methanol solution (1% (v/v), 16 h at 50°C). In contrast to Heissenberger *et al*. [49], hexane without butylated hydroxytoluene (BHT) was used for each washing step after methylation to avoid BHT-related peak interference in chromatograms. FAMEs were identified by comparison with known standards (Supelco 37 FAME Mix) using a gas chromatograph (Thermo Scientific TRACE GC Ultra™) equipped with a flame ionization detector (FID) and a Supelco™ SP-2560 column (100 m, 25 mm i.d., 0.2 µm film thickness). Quantification of FAs was performed by comparison with a known concentration of the internal standard using Excalibur 1.4™ (Thermo Electron Corporation).

### 2.9. Data analysis

All data analyses were carried out in R Studio (R v. 3.4.0) [50]. Data reported within the Results section are means ± s.e. Figures were created using ‘ggplot2’ [51]. For all boxplots, a horizontal line indicates the median, boxes indicate the interquartile range (IQR), whiskers indicate points within the 1.5 IQR and any data not included in the box and whiskers are shown as outliers (small dots). An additional point (thick black dot) was added to display the mean. Datasets that departed from normality, identified using Shapiro–Wilk tests, were transformed before analysis, except for percentage data, which were arcsine transformed. Interactions between sex and ploidy were tested using two-way ANOVAs, and comparisons between diploid and triploid Atlantic salmon were analysed using one-way ANOVA. Differences between groups were determined by post hoc Tukey's HSD test. Linear regression [50] was used to test the relationship between lipid content and the amount of FA present within the muscle tissue of triploid and diploid Atlantic salmon. A probability level of *α* = 0.05 was used to determine statistical significance. Pearson correlations were used to test the relationship between multiple behavioural recordings of sperm from individual males [50]. All recordings of sperm behaviour were identified as ‘repeatable’ (*p* < 0.001 and *r*^2^ > 0.90).

In this study, essential FAs are regarded as those that stimulate growth or any other biological response by the animal being studied, as well as FAs required for survival [52]. Principal component analysis (PCA) [50] was used to reduce the large number of individual FAs into a single FA composition score [53,54] and to analyse the difference in overall FA muscle tissue composition between treatments. To test the dependence of FAs on lipid content in both diploid and triploid Atlantic salmon, regression analysis was applied. In an effort to assess how lipids affect the composition of FAs within salmon muscle tissues, we also lipid-normalized FAs using the following equation: FA_(mg FA/g dw)_/(lipid_(mg total lipids/g dw)_/1000) [55].

## 3. Results

### 3.1. Body condition

**Figure 1.**
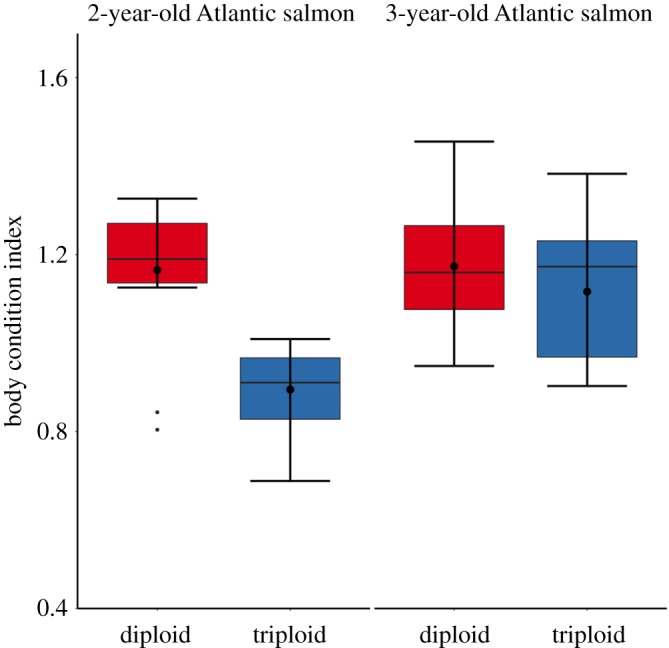
Body condition index of diploid and triploid farmed 2-year-old (*n* = 15 diploid; *n* = 15 triploid) and 3-year-old (*n* = 20 diploid; *n* = 20 triploid) Atlantic salmon reared in identical environments. Boxplots show median (horizontal black line) and mean (thick black dot); whiskers indicate points within the 1.5 IQR and quartile ranges (coloured box area).

Two-way ANOVA revealed no significant interactions between ploidy and sex in relation to body condition indices; therefore, 2- and 3-year-old diploid and triploid salmon were not categorized by sex for analysis. Despite being reared under similar conditions and on identical diets, triploid salmon (1.16 ± 0.04) (mean ± s.e.) in the 2 year age class had significantly lower body condition than the diploid salmon (0.89 ± 0.02) (*F*_1–28_ = 33.25, *p* < 0.001; figure 1). However, by year 3, triploid salmon had caught up and there was no significant difference in body condition between triploids (1.12 ± 0.03) and diploids (1.17 ± 0.03) (*F*_1–38_ = 1.68, *p* = 0.202; figure 1).

### 3.2. Primary reproductive development

**Figure 2.**
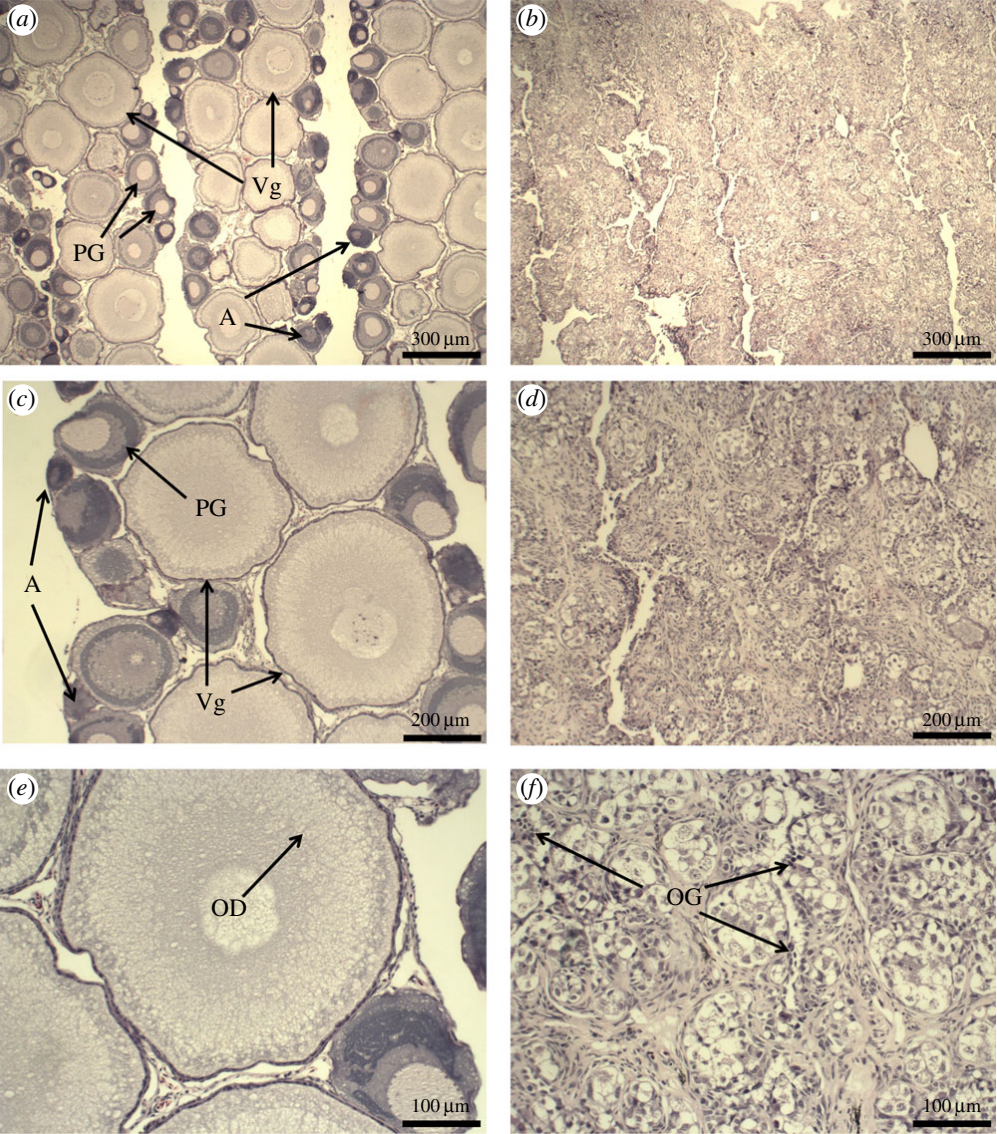
Histological sections of female diploid (*n* = 9) and triploid (*n* = 8) 2-year-old Atlantic salmon. (*a*,*c*,*e*) Diploid females containing oocytes at different stages of development (Vg, vitellogenic oocytes; PG, primary growth oocytes; A, atresia). Vitellogenic oocytes have accumulated oil droplets (OD). (*b*,*d*,*f*) Triploid females contained oogonia (OG), but ovaries are under-developed with the complete absence of maturing oocytes.

Gametogenesis was evident in all 2-year-old diploid salmon, but not in female triploids. Diploid female ovarian tissue contained both primary growth and vitellogenic oocytes. Oocyte atresia was observed, but was relatively infrequent compared with the proliferation of developing oocyte stages (figure 2). In triploid females, no primary growth or vitellogenic oocytes were observed (figure 2). Triploid ovarian tissue contained some oogonia, but no mature reproductive structures typical of ovarian development and widely observed in the diploid ovaries. The GSI of female 2-year-old triploid salmon (0.10 ± 0.01) was less than half the size of diploid females (0.25 ± 0.02) (*F*_1–15_ = 38.63, *p* < 0.001; figure 4).

**Figure 3.**
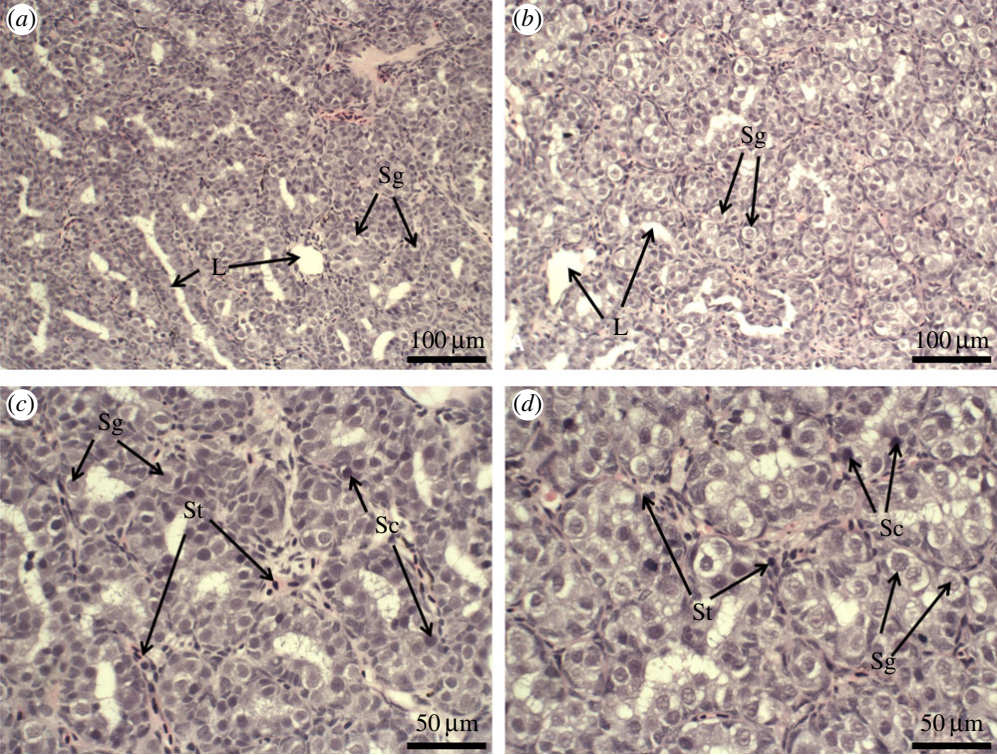
Histological sections of 2-year-old male triploid (*n* = 7) and diploid (*n* = 6) Atlantic salmon. (*a*,*c*) Diploid males showing developed gonadal structures (L, lumen) and spermatogenesis in gonads, including the presence of spermatogonia (Sg), spermatids (St) and spermatocytes (Sc). (*b*,*d*) Triploid male salmon showing similar gonadal structures to diploid salmon, including definitive aspects of spermatogenesis.

**Figure 4.**
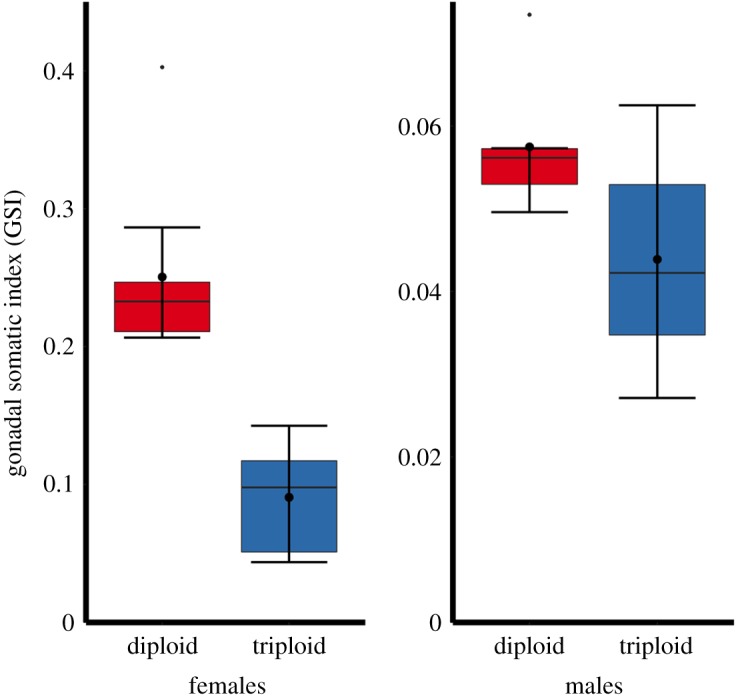
Gonadal somatic index of 2-year-old female and male triploid (*n* = 15) and diploid (*n* = 15) farmed Atlantic salmon. Boxplots show median (horizontal black line) and mean (thick black dot); whiskers indicate points within the 1.5 IQR and quartile ranges (coloured box area).

In males, developing testes were evident in both diploid and triploid 2-year-old Atlantic salmon. Gonadal tissues from both diploid and triploid salmon contained spermatocytes, spermatogonia and spermatids throughout the testicular tissue. No mature spermatozoa were observed along the testis lumen in either diploid or triploid males in these year 2 salmon (figure 3). There was no significant difference in the GSI between male triploid (0.04 ± 0.01) and diploid salmon (0.06 ± 0.00) (*F*_1–11_ = 4.79, *p* = 0.051; figure 4).

### 3.3. Reproductive maturity, gamete quality and fertility

**Figure 5.**
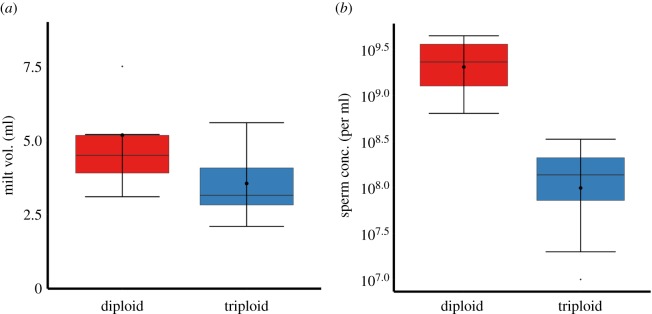
Milt volume (ml) and sperm concentration (per ml of milt) from male diploid (*n* = 10) and triploid (*n* = 10) 3-year-old Atlantic salmon. Boxplots show median (horizontal black line) and mean (thick black dot); whiskers indicate points within the 1.5 IQR and quartile ranges (coloured box area).

**Figure 6.**
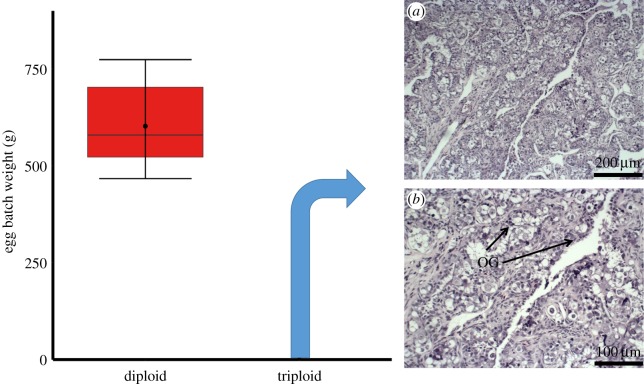
Stripped egg batch weight from female diploid Atlantic salmon (*n* = 10) and histochemical analysis of ovaries at (*a*) 100× and (*b*) 200× magnification from 3-year-old triploid salmon, showing oogonia (OG), but no evidence of mature oocytes in triploid female ovaries (*n* = 10). Boxplots show median (horizontal black line) and mean (thick black dot); whiskers indicate points within the 1.5 IQR and quartile ranges (coloured box area).

Triploid male Atlantic salmon at 3 years of age produced motile spermatozoa. Both diploid and triploid males had large quantities of strippable milt containing many millions of spermatozoa, though strips from triploid males produced less milt (3.55 ± 0.36 ml) than those from diploid males (5.18 ± 0.74 ml) (*F*_1–18_ = 51.01, *p* = 0.029; figure 5), and milt from triploid males also contained lower sperm concentrations (1.5 × 10^8^ ± 4.4 × 10^7^ sperm per ml of milt) than that from diploids (2.4 × 10^9^ ± 4.4 × 10^8^) (*F*_1–18_ = 51.01, *p* < 0.001; figure 5). Three-year-old female triploid salmon produced no eggs, while diploid females produced, on average, 602.30 ± 36.23 g of eggs per fish (figure 6). Further histochemical examinations of ovaries from 3-year-old triploid Atlantic salmon showed no signs of oogenesis. Ovaries contained no germ cells, no primary growth oocytes and no vitellogenic oocytes.

**Figure 7.**
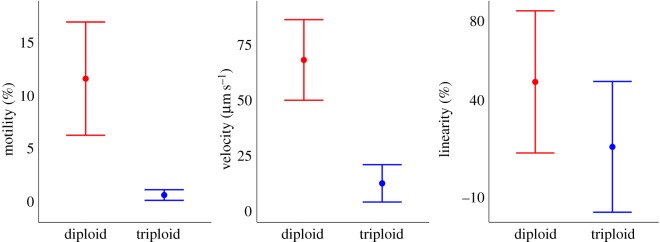
Interval plot of mean CASA measures (±s.e.) of sperm behaviour from 3-year-old male diploid (*n* = 10) and triploid (*n* = 10) Atlantic salmon.

CASA [44] measures of sperm behaviour revealed that 3-year-old triploid Atlantic salmon produced motile sperm. However, comparisons between sperm from farmed diploid and triploid Atlantic salmon showed that diploid salmon produced a significantly greater proportion of sperm that were motile (11.61 ± 5.36%) than triploid conspecifics (0.63 ± 0.5) (*F*_1–18_ = 7.25, *p* = 0.015; figure 7). Sperm produced by triploid males also had a significantly lower velocity (12.61 ± 8.43 µm s^−1^) than sperm from diploid male salmon (68.31 ± 18.19) (*F*_1–18_ = 4.99, *p* = 0.039; figure 7). Although there was a tendency for increased straightness in diploid males, there was no significant difference in the linearity of the swimming path between triploid (15.80 ± 10.5%) and diploid (48.9 ± 11.5%) salmon sperm (*F*_1–18_ = 3.28, *p* = 0.087; figure 7).

**Figure 8.**
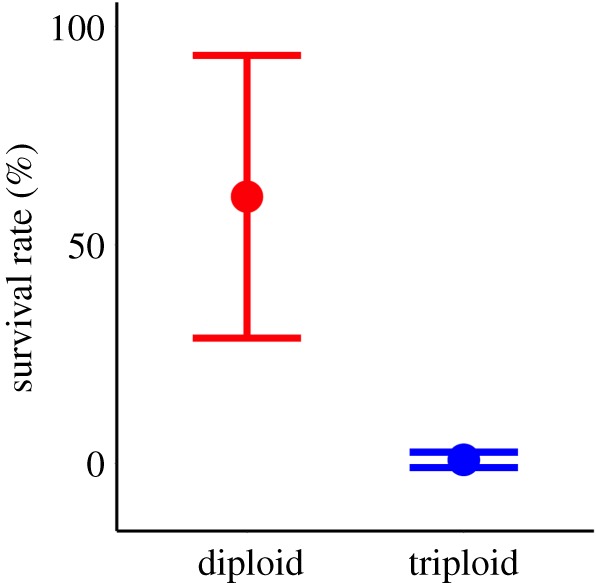
Interval plot of mean survival rates of eggs (±s.e.) to the eyed stage following *in vitro* fertilization by sperm from either diploid (*n* = 10) or triploid (*n* = 10) Atlantic salmon.

Sperm produced by triploid male salmon were capable of fertilizing ova from diploid wild female salmon. However, the mean survival rate to the eyed stage of eggs exposed to sperm from triploid males was only 1% (s.e. = 1%), compared with 61% (s.e. = 10.22) for equivalent eggs fertilized by sperm from diploid males (*F*_1–18_ = 49.09, *p* < 0.001; figure 8).

### 3.4. Fillet quality analyses

#### 3.4.1. Total lipid content and fatty acid

**Figure 9.**
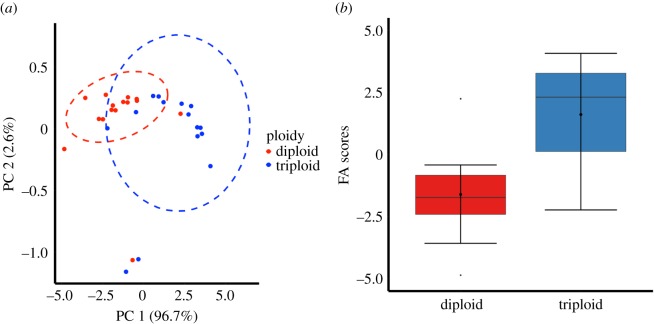
PCA of muscle tissue FA analysis with 0.95 confidence ellipses (*a*) and differences in principal component FA scores between 2-year-old diploid and triploid Atlantic salmon (*b*). Boxplots show median (horizontal black line) and mean (thick black dot); whiskers indicate points within the 1.5 IQR and quartile ranges (coloured box area).

Two-way ANOVA showed no significant interactions between ploidy and sex in relation to muscle tissue lipid content in 2-year-old Atlantic salmon. There was, however, a significant difference in the overall amount of lipid in muscle tissues between triploid and diploid Atlantic salmon (*F*_1–28_ = 16.74, *p* < 0.001), with triploid Atlantic salmon (79.94 ± 10.10 mg g dw^−1^) having significantly lower amounts of total lipids in muscle tissue than diploids (131.27 ± 7.45 mg g dw^−1^) (figure 9).

#### 3.4.2. Multivariate muscle tissue fatty acid scores of diploid and triploid salmon

**Figure 10.**
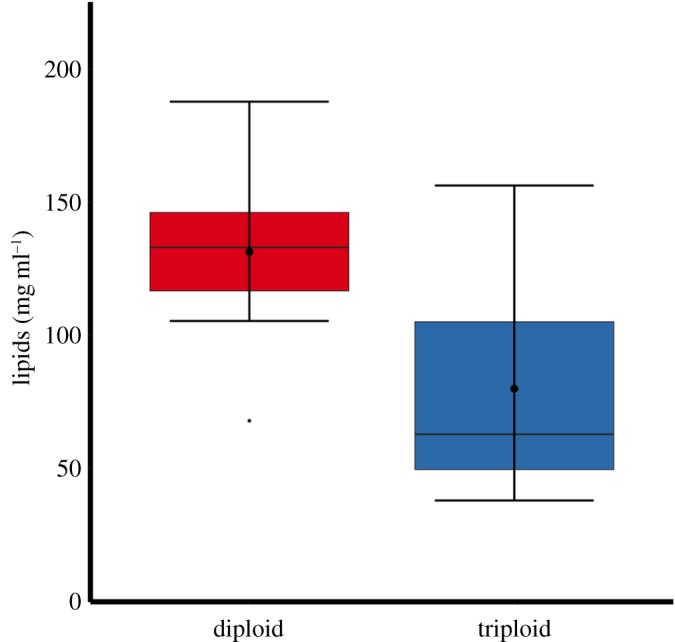
Boxplot showing muscle tissue total lipid content is higher in 2-year-old diploid (*n* = 15) than in triploid (*n* = 15) Atlantic salmon. Boxplots show median (horizontal black line) and mean (thick black dot); whiskers indicate points within the 1.5 IQR and quartile ranges (coloured box area).

The first principal component (PC 1) of the PCA, including six grouped FA variables, accounted for 96.7% of the total variance of the PCA (table 2) and showed high negative coefficients for PUFA, n-3 PUFA, SAFA, omega-6 PUFA, terrestrial fatty acids (Terr. FAs) and monounsaturated fatty acids (MUFAs) (in order of coefficient value) (table 2). The second component (PC 2) accounted for only 2.6% of the total variance of the PCA and showed a high positive coefficient for MUFAs (table 2). Using PC 1 as a model descriptor for muscle tissue overall FA content, there was a significant difference between diploid and triploid salmon muscle tissue FA compositions (*F*_1–28_ = 23.97; *p* < 0.001), with triploid salmon containing higher mean FA scores in their muscle tissue (1.67 ± 0.51) than diploid salmon (−1.61 ± 0.42) (figure 10).

**Table 2.** PCA proportion of variance and coefficients applied to each variable measured and the total variation explained (%).

content	PC 1	PC 2	PC 3	PC 4	PC 5	PC 6
proportion of variance	0.97	0.03	>0.00	>0.00	>0.00	>0.00
SAFAs	−0.41	−0.10	−0.48	−0.76	0.07	0.000
MUFAs	−0.39	0.92	0.09	0.04	0.04	0.000
Terr. FAs	−0.41	−0.13	−0.64	0.64	−0.05	0.000
PUFAs	−0.41	−0.20	0.34	0.02	−0.17	0.080
n-3 PUFAs	−0.41	−0.18	0.33	−0.02	−0.63	−0.53
n-6 PUFAs	−0.41	−0.24	0.35	0.10	−0.74	−0.27

#### 3.4.3. Total lipid content and fatty acid composition

Despite receiving the same quantity of feed over the same period of time, there were significant differences in group FA content and individual FA (mg FAME g dw^−1^) content between triploid and diploid Atlantic salmon (table 3; electronic supplementary material, table S3). Triploid Atlantic salmon contained, on average, lower FA contents in muscle tissue than diploid fish. Compared with diploids, triploid salmon contained significantly lower amounts of muscle tissue SAFAs (*F*_1–28_ = 24.74; *p* < 0.001), MUFAs (*F*_1–28_ = 26.59; *p* < 0.001), Terr. FAs (*F*_1–28_ = 22.53; *p* < 0.001) and PUFAs (*F*_1–28_ = 19.33; *p* < 0.001), as well as lower amounts of both n-3 PUFAs (*F*_1–28_ = 20.77; *p* < 0.001) and n-6 PUFAs (*F*_1–28_ = 16.53; *p* < 0.001) (table 3). This trend was also observed in individual essential FAs of tissues, with triploid salmon containing significantly lower amounts of the essential n-6 PUFA linoleic acid (LA) (*F*_1–28_ = 14.35; *p* < 0.001) and the essential n-3 PUFA α-linolenic acid (ALA) (*F*_1–28_ = 15.50; *p* < 0.001); plus significantly lower contents of arachidonic acid (ARA) (*F*_1–28_ = 27.48; *p* < 0.001), EPA (*F*_1–28_ = 16.41; *p* < 0.001) and DHA (*F*_1–28_ = 22.74; *p* < 0.001; table 3).

**Table 3.** Selected FA contents (mg FAME g dw^−1^) of muscle tissue from diploid (*n* = 15) and triploid (*n* = 15) 2-year-old Atlantic salmon (mean ± s.e.).

content	diploid	triploid	*p*-value
SAFAs	24.01 ± 1.60	11.78 ± 1.66	≤0.001
MUFAs	34.05 ± 3.12	14.21 ± 2.30	≤0.001
Terr. FAs	0.32 ± 0.03	0.14 ± 0.02	≤0.001
PUFAs	35.84 ± 1.89	22.44 ± 2.39	≤0.001
n-3 PUFAs	26.47 ± 1.25	17.34 ± 1.57	≤0.001
n-6 PUFAs	9.41 ± 0.65	5.11 ± 0.82	≤0.001
LA	7.32 ± 0.51	4.00 ± 0.69	≤0.001
ALA	2.62 ± 0.18	1.40 ± 0.24	≤0.001
ARA	0.55 ± 0.02	0.38 ± 0.02	≤0.001
EPA	5.80 ± 0.28	3.93 ± 0.36	≤0.001
DHA	13.39 ± 0.51	9.51 ± 0.62	≤0.001

#### 3.4.4. Relationships between lipid content and muscle tissue fatty acids

**Figure 11.**
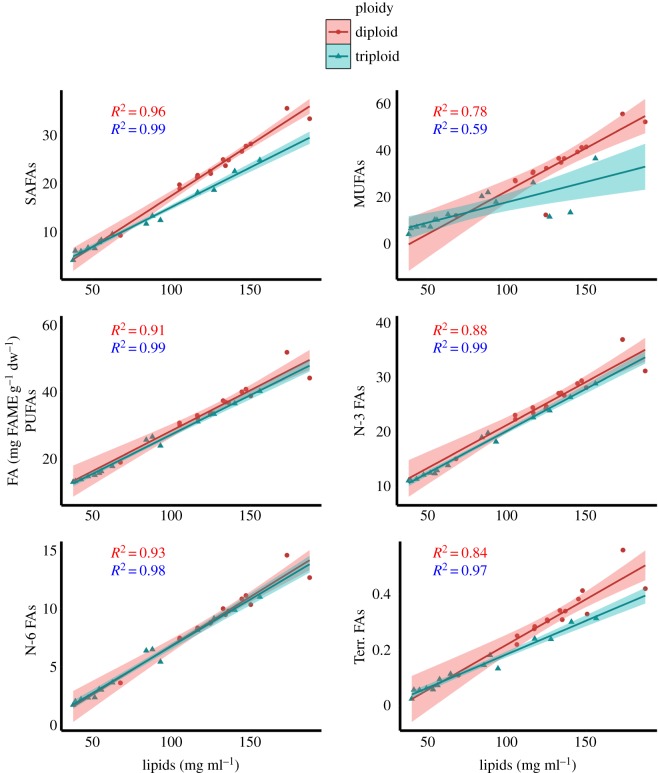
Scatterplots outlining the relationships between total lipids and groups of FAs in 2-year-old diploid (*n* = 15) and triploid (*n* = 15) Atlantic salmon muscle tissue. Linear regression (red = diploid; blue = triploid), 95% confidence intervals and *R*^2^ scores (respective colour shading).

**Figure 12.**
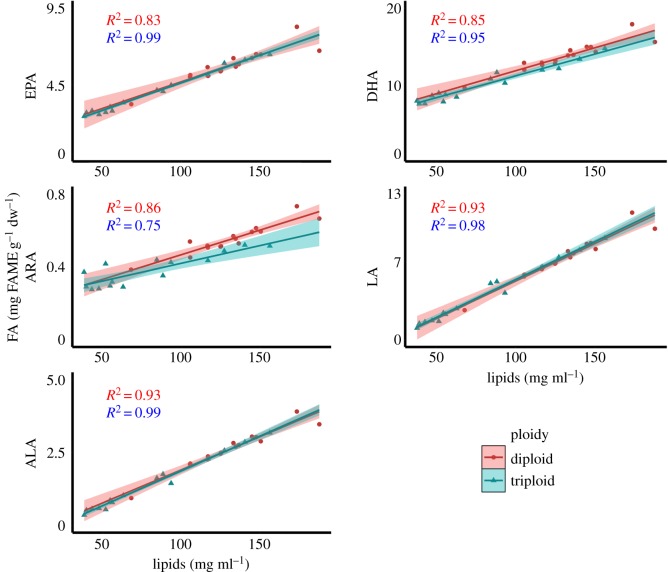
Scatterplot outlining the relationship between total lipids and selected FAs in 2-year-old diploid (*n* = 15) and triploid (*n* = 15) Atlantic salmon muscle tissue. Linear regression (red = diploid; blue = triploid), 95% confidence intervals and *R*^2^ scores (respective colour shading).

As total lipid content differed significantly between diploid and triploid Atlantic salmon muscle tissue, as well as varying more than threefold between individual fillet samples, the relationship between total lipid content and FA content in diploid and triploid salmon was examined. In grouped FAs, total lipids significantly predicted the amount of SAFAs, MUFAs, Terr. FAs, PUFAs, n-3 FAs and n-6 FAs in both diploid (*R*^2^ values ranged from 0.78 to 0.98) and triploid (*R*^2^ values ranged from 0.59 to 0.99) muscle tissue (figure 11 and table 4). Similar relationships were found in biologically important individual FAs, with total lipids significantly predicting LA, ALA, ARA, EPA and DHA contents within diploid (*R*^2^ values ranged from 0.83 to 0.93) and triploid (*R*^2^ values ranged from 0.75 to 0.99) Atlantic salmon muscle tissue (figure 12 and table 4).

**Table 4.** Linear regression model output of lipid content in muscle tissues used to predict the amount of selected FAs within the muscle tissue of 2-year-old diploid (*n* = 15) and triploid (*n* = 15) Atlantic salmon.

treatment	diploid					triploid				
	slope	intercept	*F*-value	*R*^2^-value	*p*-value	slope	intercept	*F*-value	*R*^2^-value	*p*-value
SAFAs	0.21	−3.75	345.40	0.96	<0.001	0.16	−1.33	998.1	0.99	<0.001
MUFAs	0.37	−14.46	46.00	0.78	<0.001	0.17	0.22	18.94	0.59	<0.001
Terr. FAs	0.00	−0.10	66.55	0.84	<0.001	0.00	−0.05	425.60	0.97	<0.001
PUFAs	0.24	4.12	119.20	0.91	<0.001	0.23	3.65	1071.00	0.99	<0.001
n-3 PUFAs	0.16	5.73	97.05	0.88	<0.001	0.15	4.98	1091.00	0.99	<0.001
n-6 PUFAs	0.08	−1.62	174.4	0.93	<0.001	0.08	−1.32	763.50	0.98	<0.001
LA	0.07	−1.49	178.60	0.93	<0.001	0.07	−1.42	761.10	0.98	<0.001
ALA	0.02	−0.36	174.9	0.93	<0.001	0.02	−0.51	928.90	0.99	<0.001
ARA	0.00	0.20	83.06	0.86	<0.001	0.00	0.22	39.25	0.75	<0.001
EPA	0.03	1.25	65.69	0.83	<0.001	0.04	1.07	915.20	0.99	<0.001
DHA	0.06	5.10	77.28	0.85	<0.001	0.06	4.72	226.90	0.95	<0.001

When FA content was normalized for total lipid content [55], diploid salmon continued to have higher contents of SAFAs (*F*_1–28_ = 45.23; *p* < 0.001), MUFAs (*F*_1–28_ = 14.17; *p* < 0.001), Terr. FAs (*F*_1–28_ = 24.41; *p* < 0.001), n-6 PUFAs (*F*_1–28_ = 11.87; *p* = 0.002), LA (*F*_1–28_ = 8.75; *p* = 0.007) and ALA (*F*_1–28_ = 11.11; *p* = 0.003) in their muscle tissue than triploid Atlantic salmon (table 5). By contrast, triploid salmon contained significantly higher n-3 PUFA (*F*_1–28_ = 7.05; *p* = 0.013), ARA (*F*_1–28_ = 4.90; *p* = 0.036), EPA (*F*_1–28_ = 10.96; *p* = 0.003) and DHA (*F*_1–28_ = 9.10; *p* = 0.006) contents when muscle tissue was normalized to total lipid content (table 5; electronic supplementary material, table S4).

**Table 5.** Selected lipid-normalized FAs (mg of FAs per g of total lipids) of muscle tissue from 2-year-old diploid (*n* = 15) and triploid (*n* = 15) Atlantic salmon (mean ± s.e.).

content	diploid	triploid	*p*-value
SAFAs	181.01 ± 3.67	144.00 ± 3.45	<0.001
MUFAs	253.87 ± 13.39	174.24 ± 13.30	<0.001
Terr. FAs	2.40 ± 0.09	1.58 ± 0.11	<0.001
PUFAs	274.29 ± 3.82	291.22 ± 7.30	0.063
n-3 PUFAs	203.76 ± 3.62	231.94 ± 9.19	0.013
n-6 PUFAs	70.85 ± 1.64	59.95 ± 2.79	0.002
LA	55.03 ± 1.43	45.82 ± 2.85	0.007
ALA	19.75 ± 0.49	16.00 ± 0.97	0.003
ARA	4.28 ± 0.13	5.43 ± 0.47	0.036
EPA	44.63 ± 0.88	52.35 ± 2.14	0.003
DHA	104.04 ± 2.94	132 ± 8.56	0.006

## 4. Discussion

We have confirmed that hatchery-reared male triploid Atlantic salmon produce motile spermatozoa capable of fertilizing eggs of wild female Atlantic salmon that develop to the eyed stage. Despite gametogenesis and the production of motile sperm, however, we found that male triploids showed slower maturation rates, produced fewer spermatozoa with lower motility scores and <1% of the eggs fertilized by triploid male sperm survived to the eyed stage of development. Unlike male triploids, we found that female triploid salmon exhibited no gametogenesis. Despite triploid females and males investing little or no lipids into reproductive tissues, there was no indication that additional lipids were allocated into triploid somatic muscle tissues. Consequently, we show that farm-strain triploid salmon contained less n-3 and n-6 PUFAs within their muscle tissue than farm-strain diploid salmon. However, after adjusting for muscle tissue lipid content, triploid salmon muscle tissue contained higher amounts of n-3 LC-PUFAs, ARA, EPA and DHA than diploid salmon.

### 4.1. Growth, size and condition

After 2 years of development under standard conditions, diploid Atlantic salmon had higher body condition indexes than triploids. However, by the third year of development, there was no significant difference in body condition indices. Previous studies regarding the effect of ploidy on length, weight and other body condition indices show the full spectrum of possible results [23,56,57]. Galbreath *et al*. [58] found that triploid Atlantic salmon grew faster than diploid salmon, while McGeachy *et al*. [59] observed the opposite, stating that triploid salmon at the same stage of development weighed less than diploid conspecifics for the first eight months of their life. However, similar to the results observed during the current study, McGeachy *et al*. [59] stated that weight differences between diploid and triploid salmon became insignificant over time. In a recent study, Sambraus *et al*. [60] observed that after pit tagging 1-year-old triploid and diploid Atlantic salmon and transferring them both to experimental facilities, triploid Atlantic salmon had a significantly lower body condition than diploid salmon. However, after a one-month acclimation period, during which both diploid and triploid salmon were exposed to 24 h of continuous light, triploid salmon had a significantly higher body condition than diploid salmon.

It was further suggested that triploid Atlantic salmon are more sensitive to environmental variation than their diploid counterparts [61], which could explain our findings for 2-year-old salmon. Triploid Atlantic salmon subjected to a combination of warm temperatures (19°C) and reduced oxygen saturation levels (70%), for example, exhibited lower body condition indices than diploid salmon under similar conditions [61]. By the third year, we found that triploid salmon had reached a similar size to diploids, and improved their condition scores to match the diploids, potentially adapting to hatchery conditions.

### 4.2. Reproductive function

We found that triploid female Atlantic salmon produced no gametes and, via histological examination of the ovary, showed no reproductive development towards maturation over two seasons of growth. Although Benfey [11] and Johnstone [62] observed female triploid salmonids producing some ovulated eggs, the embryos died soon after fertilization. Triploid females, generally, do not reach ovulation as most oogonia fail to proceed to the oocyte stage due to insufficient functional steroid biosynthesis [63]. Without this process, these females retain the physiological profiles of juvenile fish throughout their entire life cycle [64], with the majority of studies consequently reporting a complete lack of development in the ovaries of triploid female fish. Benfey *et al.* [65] showed that the development of ovaries within triploid pink salmon (*Oncorhynchus gorbuscha*) was severely retarded compared with diploid fish. Histological examinations of female triploid ovaries in other species show similar findings to our own, with Luo *et al*. [66] finding that the ovaries of triploid female crucian carp (*Carassius carassius*) showed no sign of vitellogenic oocytes, and Han *et al*. [67] observing no ovarian cell development in rainbow trout (*O. mykiss*). Feminization is another possible breeding management tool for farm Atlantic salmon [58,68,69]. Therefore, combining feminization and triploidy may ensure 100% sterilization of farm Atlantic salmon, preventing any hybridization with wild conspecifics. Given the complete absence of gonadal development, female triploids may also be a better choice for retaining higher amounts of dietary nutrients within muscle tissues.

Contrary to female triploid sterility, the male triploid farm salmon did have mature gonads and produced motile gametes. These findings are important, due to the fact that triploid male fish, including farm Atlantic salmon, exhibit normal spawning behaviour and attempt to fertilize eggs [27]. We found that sperm from triploid male farm salmon could fertilize eggs from diploid wild females under *in vitro* hatchery conditions, but these eggs ultimately showed a much-reduced survival rate, with <1% of the wild salmon eggs exposed to triploid milt reaching the eyed stage of development, compared with an average of greater than 60% for diploid male equivalents. Three-year-old male triploid salmon did develop enlarged testes, which were not statistically different in size from diploid male salmon, with an average gonadal somatic index of triploids being two-thirds that of diploid males. Histological sections of testicular tissue showed that both diploid and triploid male salmon contained spermatids, spermatogonia and spermatocytes. Our findings are comparable to previous studies showing similar evidence of male triploid testis development. For example, Benfey & Sutterlin [64] found that 9-month-old triploid Atlantic salmon males had a lower GSI, which was 52% that of diploids; their gonads also contained spermatids and spermatocytes.

Although triploid male salmon showed no reproductive development or function in the second year of their life cycle, they produced sperm after 3 years of development. However, triploid salmon produced less milt than diploid salmon, and the concentration of sperm per unit of milt was also an order of magnitude lower than that of diploid salmon. Despite these differences, triploid male salmon still produced hundreds of millions of sperm per strip. Sperm produced by some triploid males were motile, but motility was reduced among triploid males compared with diploid conspecifics, and the development rates of wild eggs to the eyed stage fertilized by triploid sperm were very low. Although there has not been a reported comparison within Atlantic salmon, Benfey *et al*. [70] described milt produced by triploid rainbow trout as ‘watery’ compared with that produced by diploid males. Linhart *et al*. [25] observed similar results for tench males with triploid males producing less sperm than diploids. By contrast, Peruzzi *et al.* [71] found no difference in sperm counts produced by male diploid and triploid Atlantic cod (*Gadus morhua*). It is therefore clear, from our results, that triploid induction does not eliminate male potential to produce motile sperm in Atlantic salmon, but that spermatogenic ability is reduced.

In a similar manner to sperm number, we found that triploid males produced sperm showing reduced motility. Sperm motility can be closely linked to fertilization and sperm competition success in fishes [21], but it is rarely examined when assessing triploid function. The few existing studies parallel our own findings for reduced sperm motility in triploid males. Spermatozoa recovered from triploid tench were 23–56% slower than those from diploid males [25]. Previous studies have suggested that the reason behind poor motility characteristics in sperm produced by triploid fish is due to genetic and structural abnormalities [72]. For example, detailed gene expression analyses of diploid and triploid cyprinid testes observed under-expression of dynein and intraflagellar transport genes (DNAHs, DNAL1, IFTs and DNAAF1) in triploids; these genes are associated with sperm flagellum assembly and motility [72]. These disrupted spermatogenic pathways in triploids may also lead to an increased prevalence of sperm abnormalities, as recorded in triploid masu salmon (*Oncorhynchus masou*), which produced large numbers of abnormal biflagellate or bicephalic sperm [73].

Given the reduced number and motility of what are likely to be aneuploid sperm [11], our egg survival rates from triploid males were predictably low, with <1% of eggs exposed to triploid male sperm reaching the later eyed stage of development. These findings are consistent with poor embryonic development in eggs created by crosses between triploid male sperm and diploid female eggs in a number of other species (see also [11]). Peruzzi *et al*. [71], for example, showed that, despite producing similar numbers of sperm to diploid conspecifics, fertilization of eggs by triploid male Atlantic cod was reduced (12% compared with 63%). The low egg survival rates of eggs fertilized by triploid males reported within the current study may be due to differences in the number of chromosomes present in triploid sperm. Spermatozoa from triploids are generally aneuploid, containing abnormal numbers of chromosomes compared with normal haploid gametes and, when these sperm fertilize eggs, the subsequent embryos and larvae have abnormal ploidy levels that are intermediate between diploid and triploid conditions [11]. Larvae produced using triploid Atlantic cod sperm showed a mean ploidy level around 2.4n, ranging from nearly diploid (2.1n) to almost triploid (2.75n) [71]. These aneuploid larvae suffered developmental abnormalities and died shortly after hatching.

### 4.3. Fillet quality

Fish are the major source of n-3 LC-PUFAs such as EPA and DHA for humans, with Atlantic salmon containing particularly high contents [1]. Omega-3 PUFAs have a wide range of physiological roles linked to human health [1], such as in decreasing risks of cardiovascular disease, improving effectiveness of chemotherapeutic agents during cancer therapy and mitigating inflammatory conditions, such as rheumatoid arthritis, irritable bowel disease and asthma [2]. Aquaculture currently supplies the world with the majority of its dietary EPA and DHA [1]. Unfortunately, the amount of dietary DHA and EPA contained in farmed Atlantic salmon diets has fallen, leading to a decrease in the nutritional quality of farmed salmon [1]. Owing to global change and increasing water temperatures, dietary supply of EPA and DHA is expected to decline even further worldwide [74]. Absolute contents of EPA and DHA in the muscle tissue of farmed Atlantic salmon, for example, have fallen significantly in recent years from 2.74 g per 100 g wet weight in 2006 to 1.36 g in Scottish salmon farms and 1.00 g in Norwegian salmon farms during 2013 [1,75]. Generally, the World Health Organization (WHO) recommends a weekly intake per person of 2–3 g of DHA + EPA (WHO 2018; see http://www.who.int/nutrition/topics/5_population_nutrient/en/index13.html), indicating that people now need to consume two to three times as much salmon to obtain equivalent benefits from these essential nutrients as they did 12 years ago.

It has been hypothesized that triploid farm fish could help solve these nutritional constraints by accumulating and storing more essential nutrients from their diet in their somatic tissue, because of a reduced requirement from normal breeding development to divert nutrients into costly reproductive maturation [18,20,76,77]. In general, we did not find this to be the case when comparing triploid and diploid farm salmon maintained under similar hatchery conditions. Triploid salmon of both sexes had significantly lower lipid content within muscle tissue than diploids, even during the breeding season. Recent studies examining whole-body lipid content between triploid and diploid Atlantic salmon have observed that triploid salmon contain significantly higher amounts of whole-body lipid than diploid counterparts [78–81]. However, comparative data regarding the lipid content of diploid and triploid Atlantic salmon muscle tissues are more scarce, with the majority of previous studies reporting muscle lipid content in other salmonid species. Results within these previous studies also contradict our current findings. For example, Cleveland *et al*. [18] and Sheehan *et al*. [82] reported no measurable discrepancies in muscle tissue lipid content between triploid and diploid rainbow trout, and similar results have been observed within a variety of species, such as goldfish (*Carassius auratus*) and Nile tilapia (*Oreochromis niloticus*) [83,84].

In this study, triploid Atlantic salmon muscle tissue contained a significantly different FA profile from that of diploid equivalents. When the data were examined in detail, however, fillet analyses showed that triploid salmon muscle tissue contained significantly lower levels of every FA recorded. Previous research comparing FA content in triploid and diploid fish reveals contrasting results. Cleveland *et al*. [76] found that muscle tissue from reproductively mature triploid rainbow trout contained significantly higher amounts of SAFAs than triploid trout of the same age, suggesting that triploids retain lipid stores in the absence of sexual maturation. However, there was no significant difference in the amount of n-3 LC-PUFAs present in diploid and triploid trout muscle tissue. Alternatively, Qin *et al*. [85] reported that triploid whitespotted clarias (*Clarias fuscus*) had higher amounts of n-6 and n-3 LC-PUFAs than diploids. Both lipids and fatty acids serve a variety of important biological functions in fish. Omega-3 LC-PUFAs, in particular, provide structural composition within cell membranes and facilitate somatic growth and development [86]. Our findings, however, indicate that triploidy does not improve Atlantic salmon fillet quality, with regard to overall FA content.

Despite this overall reduction, the significant positive correlations between lipid content and the level of specific FAs present within triploid salmon muscle tissues yielded some unexpected findings. After adjusting for muscle tissue lipid content, which varied more than threefold among all fish in our samples, triploid salmon contained higher amounts of total n-3 LC-PUFAs, ARA, EPA and DHA per g of lipid than diploid salmon. The fact that triploids can bioaccumulate some PUFA content at higher levels provides an opportunity for targeted selection or farming practice to improve this potentially advantageous triploid trait. It is known that fish in particular are able to preferentially retain and endogenously synthesize EPA and DHA when dietary supplies are limited, with Murray *et al*. [54] observing that Arctic charr (*Salvelinus alpinus*) fed low contents of EPA and DHA retained higher amounts of both dietary nutrients within their muscle tissues, and increasing EPA and DHA contents being synthesized endogenously within the liver.

## 5. Conclusion

Our study confirms that spermatogenesis and some reproductive and fertile function are present within triploid farm male Atlantic salmon, but no gametogenesis was observed in female triploid salmon. Despite evidence of some male reproductive function, we conclude that triploid salmon are effectively sterile, being unable to fertilize eggs that reach the eyed stage of development in any consequential numbers. Combined with evidence that triploid fish demonstrate reduced migration to freshwater [87], we therefore conclude that triploidization will minimize reproduction by escaped farmed Atlantic salmon and therefore effectively prevent genetic introgression into wild populations.

The reduced or absent reproductive activity within triploid farm salmon was not associated with increased fillet quality. We found that triploid muscle tissue contained reduced total lipid content, and consequently lower absolute contents of SAFAs, MUFAs, n-6 LC-PUFAs and n-3 LC-PUFAs. There is, however, hope for nutritional improvement because triploid salmon were able to accumulate higher relative amounts of biologically important EPA and DHA. If targeted selection and improvement to husbandry procedures enable triploid Atlantic salmon to accrue lipid deposits to the same extent as their diploid counterparts, the nutritional quality of their fillets, in terms of essential FAs, may exceed that of the diploid salmon.

## Supplementary Material

Supplementary Table 1

## Supplementary Material

Supplementary Table 2

## Supplementary Material

Supplementary Table 3

## Supplementary Material

Supplementary Table 4

## Data accessibility

 Our data were deposited at Dryad: (http://dx.doi.org/10.5061/dryad.bt6616g) [88].
