# Supplementary material for: Comparisons of reproductive function and fatty acid fillet quality between triploid and diploid farm Atlantic salmon (Salmo salar)
Source: R Soc Open Sci. 2018 Aug 15;5(8):180493. doi: 10.1098/rsos.180493 (PMC6124059; doi:10.1098/rsos.180493)
Supplement: Supplementary Table 1 [file rsos180493supp1.pdf]

Supplementary Table 1*: List of microsatellite markers displaying three alleles for triploid salmon sampled during this study*

Sample ID	*SSsp3016*	*SSsp2210*	*SSspG7*	*SSsp2201*	*SSsp1605*	*SSsp2216*	*Ssa197*	*Ssa171*	*Ssa202*	*SsaD157*	*SsaD486*	*SsaD144*	*Ssa289*	*Ssa14*	*SsaF63*	*SsaOsl85*	*MHC I*	*MHC II*	***Total***
2015-2016																			
**Triploid 1**						X			X	X									3
**Triploid 2**	X	X				X			X				X						5
**Triploid 3**		X						X		X			X						4
**Triploid 4**	X					X			X	X									4
**Triploid 5**		X				X			X										3
**Triploid 6**	X	X				X							X					X	5
**Triploid 7**	X	X				X	X		X								X	X	7
**Triploid 8**	X	X				X	X			X								X	6
**Triploid 9**						X	X											X	3
**Triploid 10**	X				X		X		X	X					X		X		7
**Triploid 11**									X	X									2
**Triploid 12**		X			X				X									X	4
**Triploid 13**		X				X			X									X	4
**Triploid 14**						X			X	X									3
**Triploid 15**		X			X				X							X			4
**2016-2017**																			
**Triploid 1**	X				X	X		X	X								X		6
**Triploid 2**	X	X			X	X											X		5
**Triploid 3**		X						X	X										3
**Triploid 4**	X	X				X	X		X						X				6
**Triploid 5**	X	X				X	X												4
**Triploid 6**					X	X	X		X										4
**Triploid 7**	X	X				X													3
**Triploid 8**	X	X			X	X		X							X				6
**Triploid 9**		X				X									X		X		4
**Triploid 10**	X	X			X	X			X						X		X		7
**Triploid 11**	X	X				x				X								X	5
**Triploid 12**		X							X	X								X	4
**Triploid 13**		X				x			X							X		X	5
**Triploid 14**		X			X		X		X								X		5
**Triploid 15**	X	X					X											X	4
