# Supplementary material for: Comparisons of reproductive function and fatty acid fillet quality between triploid and diploid farm Atlantic salmon (Salmo salar)
Source: R Soc Open Sci. 2018 Aug 15;5(8):180493. doi: 10.1098/rsos.180493 (PMC6124059; doi:10.1098/rsos.180493)
Supplement: Supplementary Table 2 [file rsos180493supp2.pdf]

Supplementary Table 2*:* Fatty acids present in feed components. Feed was sampled twice and an average was calculated (mg FAME/g dw).

Fatty acid	Content (mg FAME/g dw)
**14:0**	7.87±0.12
**15:0**	0.46±0.01
**16:0**	15.70±0.27
**16:1n-9**	0.21±0.00
**16:1n-7**	7.52±0.12
**17:0**	0.45±0.01
**17:1n-7**	0.00±0.00
**18:0**	3.19±0.05
**18:1n-9t**	0.09±0.01
**18:1n-9c**	22.76±0.31
**18:2n-6t**	0.09±0.01
**18:2n-6c (LIN)**	10.09±0.21
**20:0**	0.46±0.01
**18:3n-6**	0.14±0.00
**20:1n-9**	10.61±0.10
**18:3n-3 (ALA)**	3.66±0.07
**21:0**	0.02±0.05
**18:4n-3**	3.13±0.04
**20:2n-6**	0.41±0.01
**22:0**	0.90±0.01
**20:3n-6**	0.13±0.00
**22:1n-9**	1.05±0.01
**20:3n-3**	0.35±0.01
**20:4n-6**	0.46±0.01
**23:0**	0.10±0.01
**20:4n-3**	0.91±0.01
**22:2n-6**	None detected
**24:0**	0.00±0.00
**20:5n-3 (EPA)**	9.02±0.01
**24:1n-9**	0.78±0.00
**22:3n-3**	0.06±0.00
**22:4n-6**	0.06±0.01
**22:5n-3**	1.19±0.01
**22:6n-3 (DHA)**	9.41±0.02

Values are mean±S.E
