# Supplementary material for: Comparisons of reproductive function and fatty acid fillet quality between triploid and diploid farm Atlantic salmon (Salmo salar)
Source: R Soc Open Sci. 2018 Aug 15;5(8):180493. doi: 10.1098/rsos.180493 (PMC6124059; doi:10.1098/rsos.180493)
Supplement: Supplementary Table 3 [file rsos180493supp3.pdf]

Supplementary Table 3: Total lipid and fatty acid contents (mg FAME/g dw) of muscle tissue from 2-year old diploid and triploid Atlantic salmon.

Content	Diploid	Triploid	P-value
**14:0**	3.47±0.26	1.57±0.31	p≤ 0.001
**15:0**	0.34±0.02	0.16±0.03	p≤ 0.001
**16:0**	16.05±1.03	7.97±1.09	p≤ 0.001
**16:1n-9**	0.25±0.02	0.12±0.02	p≤ 0.001
**16:1n-7**	4.39±0.33	2.02±0.38	p≤ 0.001
**17:0**	0.34±0.02	0.17±0.03	p≤ 0.001
**17:1n-7**	None detected	None detected	NA
**18:0**	3.39±0.25	1.70±0.19	p≤ 0.001
**18:1n-9**	0.08±0.01	0.03±0.01	p≤ 0.001
**18:1n-9c**	20.71±2.28	8.09±1.78	P= 0.171
**18:2n-6t**	0.03±0.00	0.01±0.00	P= 0.005
**18:2n-6c (LIN)**	7.32±0.52	4.00±0.69	p≤ 0.001
**20:0**	0.17±0.01	0.08±0.01	p≤ 0.001
**18:3n-6**	0.10±0.01	0.04±0.01	p≤ 0.001
**20:1n-9**	3.96±0.30	1.90±0.34	p≤ 0.001
**18:3n-3 (ALA)**	2.62±0.18	1.40±0.24	p≤ 0.001
**21:0**	0.02±0.00	0.01±0.00	p≤ 0.001
**18:4n-3**	1.04±0.07	0.53±0.10	p≤ 0.001
**20:2n-6**	0.88±0.06	0.41±0.07	p≤ 0.001
**22:0**	0.12±0.01	0.05±0.01	p≤ 0.001
**20:3n-6**	0.21±0.02	0.12±0.01	p≤ 0.001
**22:1n-9**	0.44±0.03	0.21±0.04	p≤ 0.001
**20:3n-3**	0.33±0.02	0.15±0.03	p≤ 0.001
**20:4n-6**	0.55±0.02	0.38±0.02	p≤ 0.001
**23:0**	0.08±0.00	0.06±0.04	P= 0.003
**20:4n-3**	1.07±0.07	0.57±0.08	p≤ 0.001
**22:2n-6**	0.18±0.01	0.08±0.01	p≤ 0.001
**24:0**	0.03±0.00	0.01±0.00	p≤ 0.001
**20:5n-3 (EPA)**	5.80±0.28	3.93±0.36	p≤ 0.001
**24:1n-9**	0.56±0.03	0.32±0.04	p≤ 0.001
**22:3n-3**	0.04±0.00	0.02±0.00	P= 0.003
**22:4n-6**	0.09±0.01	0.05±0.01	p≤ 0.001
**22:5n-3**	2.18±0.13	1.22±0.15	p≤ 0.001
**22:6n-3 (DHA)**	13.39±0.51	9.51±0.62	p≤ 0.001

Values are mean±S.E.
