# Supplementary material for: Comparisons of reproductive function and fatty acid fillet quality between triploid and diploid farm Atlantic salmon (Salmo salar)
Source: R Soc Open Sci. 2018 Aug 15;5(8):180493. doi: 10.1098/rsos.180493 (PMC6124059; doi:10.1098/rsos.180493)
Supplement: Supplementary Table 4 [file rsos180493supp4.pdf]

Supplementary Table 4:. Lipid-normalised fatty acids (mg of fatty acids/g of total lipids) of muscle tissue from 2-year old diploid and triploid Atlantic salmon.

Content	Diploid	Triploid	P-value
**14:0**	25.95±0.86	17.32±1.48	P < 0.001
**15:0**	2.57±0.07	1.88±0.11	P < 0.001
**16:0**	121.28±2.33	98.33±2.15	P < 0.001
**16:1n-9**	1.85±0.06	1.36±0.07	P < 0.001
**16:1n-7**	32.77±1.01	22.32±1.85	P < 0.001
**17:0**	2.55±0.06	2.09±0.07	P < 0.001
**17:1n-7**	None detected	None detected	NA
**18:0**	25.50±0.55	21.96±0.58	P < 0.001
**18:1n-9**	0.58±0.02	0.56±0.18	P = 0.437
**18:1n-9c**	153.83±12.04	103.55±12.87	P = 0.372
**18:2n-6t**	0.24±0.01	0.25±0.03	P = 0.563
**18:2n-6c (LIN)**	55.03±1.43	45.82±2.86	P= 0.007
**20:0**	1.29±0.04	0.89±0.06	P < 0.001
**18:3n-6**	0.70±0.03	0.50±0.04	P = 0.031
**20:1n-9**	29.69±0.89	21.64±1.54	P < 0.001
**18:3n-3 (ALA)**	19.75±0.49	16.00±0.47	P = 0.003
**21:0**	0.18±0.01	0.09±0.01	P < 0.001
**18:4n-3**	7.82±0.23	5.91±0.50	P = 0.003
**20:2n-6**	6.60±0.19	4.81±0.24	P < 0.001
**22:0**	0.92±0.04	0.60±0.04	P < 0.001
**20:3n-6**	1.60±0.04	1.59±0.11	P = 0.683
**22:1n-9**	3.28±0.09	2.39±0.18	P < 0.001
**20:3n-3**	2.51±0.07	1.80±0.16	P = 0.015
**20:4n-6**	4.28±0.13	5.43±0.47	P = 0.036
**23:0**	0.59±0.01	0.75±0.04	P < 0.001
**20:4n-3**	4.28±0.20	5.43±0.23	P < 0.001
**22:2n-6**	1.35±0.04	0.93±0.08	P < 0.001
**24:0**	0.20±0.01	0.20±0.02	P = 0.741
**20:5n-3 (EPA)**	44.63±0.89	52.35±2.14	P = 0.003
**24:1n-9**	4.25±0.07	4.03±0.08	P = 0.028
**22:3n-3**	0.29±0.01	0.30±0.03	P = 0.947
**22:4n-6**	0.71±0.20	0.62±0.03	P = 0.003
**22:5n-3**	16.61±0.33	15.52±0.68	P = 0.106
**22:6n-3 (DHA)**	104.04±2.94	132.71±8.56	P = 0.006

Values are mean±S.E.
